# Long non‐coding RNA NR2F1‐AS1 promoted proliferation and migration yet suppressed apoptosis of thyroid cancer cells through regulating miRNA‐338‐3p/*CCND1* axis

**DOI:** 10.1111/jcmm.14386

**Published:** 2019-07-14

**Authors:** Feng Guo, Qingfeng Fu, Yang Wang, Guoqing Sui

**Affiliations:** ^1^ Department of Ultrasound China‐Japan Union Hospital of Jilin University Changchun Jilin China; ^2^ Jilin Provincial Key Laboratory of Surgical Translational Medicine, Division of Thyroid Surgery China‐Japan Union Hospital of Jilin University Changchun Jilin China

**Keywords:** *CCND1*, miRNA‐338‐3P, NR2F1‐AS1, thyroid cancer

## Abstract

Thyroid cancer (TC) is a prevalent endocrine malignant cancer whose pathogenic mechanism remains unclear. The aim of the study was to investigate the roles of long non‐coding RNA (lncRNA) NR2F1‐AS1/miRNA‐338‐3P/*CCND1* axis in TC progression. Differentially expressed lncRNAs and mRNAs in TC tissues were screened out and visualized by R program. Relative expression of NR2F1‐AS1, miRNA‐338‐3p and cyclin D1 (*CCND1*) was determined by quantitative real time polymerase chain reaction. In addition, Western blot analysis was adopted for evaluation of protein expression of *CCND1*. Targeted relationships between NR2F1‐AS1 and miRNA‐338‐3p, as well as miRNA‐338‐3p and *CCND1* were predicted using bioinformatics analysis and validated by dual‐luciferase reporter gene assay. Besides, tumour xenograft assay was adopted for verification of the role of NR2F1‐AS1 in TC in vivo. NR2F1‐AS1 and *CCND1* were overexpressed, whereas miRNA‐338‐3p was down‐regulated in TC tissues and cell lines. Down‐regulation of NR2F1‐AS1 and *CCND1* suppressed proliferation and migration of TC cells yet greatly enhanced cell apoptotic rate. Silence of NR2F1‐AS1 significantly suppressed TC tumorigenesis in vivo. NR2F1‐AS1 sponged miRNA‐338‐3p to up‐regulate *CCND1* expression to promote TC progression. Our study demonstrated that up‐regulation of NR2F1‐AS1 accelerated TC progression through regulating miRNA‐338‐3P/*CCND1* axis.

## 1 INTRODUCTION

Thyroid cancer (TC) is the most prevalent endocrine malignancy accounting for nearly one‐third of head and neck malignancy globally.^1^ The past decades have witnessed increased morbidity and mortality of thyroid cancer globally.^2^, ^3^ There are a great number of risk factors which are closely related with TC initiation and progression, such as genetic factors, environmental exposure and epigenetic alteration.^4^ Despite improvement in detection and surgical management, including surgical resection, radiotherapy and levothyroxine treatment, more optimal therapies have remained to be further developed.^5^, ^6^ Hence, it is significant to unveil underlying mechanism of TC progression and pathogenesis, which may well provide us with more integrated and upgraded therapies for TC patients.

Long non‐coding RNAs (lncRNAs) are non‐coding polyadenylated RNAs typically longer than 200 base pairs and act as natural antisense transcripts in animals and humans.^7^ Long non‐coding RNA serves as one of vital epigenetics regulatory mechanism, as well as DNA methylation and genomic imprinting.^8^ A great number of studies have revealed that lncRNAs can take part in massive biological processes, including transcriptional activation and interference, cellular differentiation, embryogenesis and stem cell biology.^9^, ^10^ Besides, dysfunction of lncRNAs has been clarified to be associated with progression of various diseases, such as lung cancer, liver cancer and breast cancer.^11^, ^12^, ^13^ It was reported that some lncRNAs play pivotal roles in TC tumorigenesis, such as lncRNA CASC2 and lncRNA PROX1‐AS1.^14^, ^15^ Furthermore, previous reports showed that lncRNA NR2F1‐AS1 was able to regulate hepatocellular carcinoma oxaliplatin resistance by regulating miR‐363/*ABCC1* axis.^8^ Nonetheless, it remains to be demonstrated whether and how NR2F1‐AS1 functions in TC progression.

MicroRNAs are 18‐23 nucleotide‐long non‐coding (ncRNAs) RNAs that always serve as negative regulators of translation and are involved in cell functions via binding to the 3′‐untranslated region (UTR) of their targets.^16^ In addition, dysregulation of miRNAs closely related to diseases, such as cancers, cardiovascular disease and diabetes.^17^ In addition, studies revealed that some miRNAs, such as miR‐520a‐3p, miR‐718 and miR‐335‐5p are closely related with progression of TC.^18^, ^19^, ^20^ In addition, previous reports revealed that miR‐338‐3p participated in the progression of cervical cancer, colorectal cancer and renal cancer.^21^, ^22^, ^23^ However, there are no sufficient evidences of roles of miR‐338‐3p, as well as the relationship between NR2F1‐AS1 and miR‐338‐3p in the progression of TC.

Cyclin D1 (*CCND1*), one of the highly conserved members of the cyclin family, was well characterized by a periodicity in protein abundance in cell cycle.^24^ Dysfunction of *CCND1* was closely related with progression of several cancers by causing abnormal proliferation.^24^ Several reports showed the targeted relationships between *CCND1* and miRNAs in cancer. Liu et al showed that miR‐138 inhibited nasopharyngeal carcinoma growth and tumorigenesis by targeting *CCND1*. Besides, Cai et al found that *CCND1*, *CDK2* and *CDK6* could be directly targeted by miR‐186 in lung adenocarcinoma.^25^ However, the roles of *CCND1*, as well as miR‐338‐3p/*CCND1* axis in the TC tumorigenesis were little understood.

Competing endogenous RNA (ceRNA) mechanism proposed that transcripts such as mRNAs, pseudogenes and lncRNAs can serve as natural miRNA sponges by competitive binding to miRNA response elements to suppress their expression and function.^26^ However, the ceRNA mechanism in TC still needs further study.

Our study is aimed to unveil the relationship between NR2F1‐AS1 and miR‐338‐3p and their roles in TC. Our study revealed that dysregulation of NR2F1‐AS1 and miR‐338‐3p affected the TC progression via targeting *CCND1*, which might well offer us with more potential therapeutic strategies for TC.

## 2 MATERIALS AND METHODS

### 2.1 Microarray analysis and co‐expression network analysis

Microarray data were obtained from GEO (https://www.ncbi.nlm.nih.gov/geo/query/). The series accession number of both lncRNA and mRNA expression profile was GSE3678 and matched platform was GPL570. In brief, data were processed for selecting differentially expressed both lncRNAs and mRNAs using selecting criteria adj *P* value <0.05 and |FC (fold change)| > 2 by limma package. Top differentially expressed ones were visualized by heatmap. Gene set enrichment analysis (GSEA) was made using GSEA v3.0 software and was adopted to perform gene set enrichment analysis. Co‐expression network analysis was carried out based on the correlation coefficient which was calculated between differentially expressed lncRNAs and mRNAs. In brief, to establish lncRNA/mRNA co‐expression network, ‘pearson’ in ‘psych’ package was employed for validating the correlations in the selected mRNAs and lncRNAs. The networks were then adjusted by ‘BH’. Thereafter, they were graphed by Cytoscape software. In co‐expression networks, nodes represented differently expressed genes, and the edges represented the co‐expression status.

### 2.2 Clinical samples

Human specimens including 25 TC tissues and paired adjacent normal tissues were collected from 1 August 2017 to 31 March 2018 from the China‐Japan Union Hospital of Jilin University. Clinical data collection met ethical requirements, and both clinical data collection and the procedures in the study were scrutinized and approved by Medical Ethics Committee of China‐Japan Union Hospital of Jilin University. In addition, TC donors involved in the study signed their informed consent allowing the use of their tissue samples for scientific research.

### 2.3 Cell culture

FTC‐133 and Human papillary thyroid carcinoma derived cell line (B‐CPAP and one normal thyroid cell line Nthy‐ori 3‐1 were all provided by BeNa Culture Collection (Beijing, China), which were cultivated at 37°C in a humidified atmosphere of 5% CO_2_. Nthy‐ori 3‐1 cells were cultured using F‐12K medium. Besides, FTC‐133 cells and HEK‐293 cells were in DMEM medium with 90% high glucose. B‐CPAP cells were cultured in Dulbecco's Modified Eagle's Medium/Ham's F‐12 Medium (DMEM/F12) Medium. Media in the study were bought from Gibco and all supplemented with 10% of fetal bovine serum.

### 2.4 Plasmid construction and cell transfection

ShRNA negative control (NC), sh‐NR2F1‐AS1, sh‐*CCND1*, miR‐338‐3p mimics, miR‐338‐3p inhibitor and NC were all provided by GenePharma Co, Ltd (Shanghai, China). In brief, sh‐NR2F1‐AS1 was synthesized and loaded into the pENTR^TM^/U6 vector. After finishing this construction, pENTR^TM^/U6‐sh‐NR2F1‐AS1 was sequenced to verify the accuracy of the inserted sequence. Thereafter, pENTR^TM^/U6‐sh‐NR2F1‐AS1 was introduced into FTC‐133 cells and B‐CPAP cells using Lipofactamine2000 (Invitrogen, Carlsbad, CA). Stably transfected cell with antibiotic resistance were sifted and enriched by adding puromycin to culture medium. Based on protocols of Lipofectamine 2000 (Invitrogen), miR‐338‐3p mimics, miR‐338‐3p inhibitor and NC were introduced into FTC‐133 cells and B‐CPAP cells. PcDNA3.1‐NR2F1‐AS1 and pcDNA3.1‐*CCND1* were from GenePharma as well. Before plasmid transfection, FTC‐133 cells and B‐CPAP cells were suspended and seeded in six‐well culture plates at 37°C. When cell confluence rate reached 80% to 90%, cell culture media should be replaced by serum‐free fresh medium 3 hours before transfection.

### 2.5 Protein isolation and Western blot analysis

Proteins were isolated by radio‐immunoprecipitation assay buffer and protein concentration was determined using bicinchoninic acid (BCA) protein concentration assay Kit (Abcam). Thereafter, protein samples subjected to SDS‐PAGE ran for 4 hours. Gel‐separated proteins were subsequently transferred to polyvinylidene fluoride membranes, and exposed to 5% non‐fat milk overnight incubation with the rabbit polyclonal anti‐GAPDH (ab181602, 1:5000; Abcam), rabbit polyclonal anti‐CCND1 (ab226977, 1:10000; Abcam) at 4°C. Afterwards, membranes were washed by tris‐buffered saline and then incubated with second antibody horseradish peroxidase (HRP)‐conjugated goat‐anti‐rabbit IgG (ab205718, 1:2000; Abcam) for about 3 hours.

### 2.6 qRT‐PCR

Total RNA was extracted using TRIzol^TM^ Reagent. Afterwards, Reverse Transcription Kit was served for reverse transcription of the RNA into cDNA. Quantitative real time polymerase chain reaction (qRT‐PCR) was carried out through SuperReal SYBR Green kit. Normalization of mRNAs and lncRNAs expression values was performed against GAPDH, respectively. A TaqMan miRNA assay kit was obtained from Thermal Fisher Scientific. miRNAs expression was normalized against U6. All of relative expression value was quantified by $2^{-\Delta \Delta C_{t}}$ method and primers for qRT‐PCR were all enlisted in Table 1.

**Table 1:** Primers in this study

Gene	Primers
NR2F1‐AS1‐F	CAGCGGTGCAAACCATGTGC
NR2F1‐AS1‐R	GTAAACCAAGTCGGTTGAACG
CCND1‐F	CGTGGCCTCTAAGATGAAGG
CCND1‐R	CTGGCATTTTGGAGAGGAAG
GAPDH‐F	CAGTGCCAGCCTCGTCTAT
GAPDH‐R	CTTCTGACACCTACCGGGGA
miR‐338‐3p‐F	TGCGGTCCAGCATCAGTGAT
miR‐338‐3p‐R	TGGAGCCTGGGACGTGACC
U6‐F	TGCGGGTGCTCGCTTCGGCAGC
U6‐R	CCAGTGCAGGGTCCGAGGT

### 2.7 Cell Counting Kit‐8 Assay

Cell proliferation ability was evaluated by Cell Counting Kit‐8 (CCK‐8; Engreen, Beijing, China). In brief, cells were placed in 96‐well plates and pre‐incubated with 15 μL CCK‐8 solutions in each well. Optical density (OD) at 450 nm wavelength was measured every 24 hours after transfection.

### 2.8 Dual‐luciferase reporter gene assay

Targeted relationships between miRNAs and mRNAs were predicted using TargetScan (http://www.targetscan.org/vert_/72) and binding sites between miRNAs and lncRNAs were predicted using miRcode (http://www.mircode.org/index.php). PmirGLO luciferase reporter gene vector with NR2F1‐AS1‐wt or NR2F1‐AS1‐mut was introduced into HEK‐293 cells with co‐transfection of miR‐338‐3p mimics or control using Lipofectamine 2000 reagent (Invitrogen). Similarly, luciferase reporter gene vector with *CCND1*‐wt or *CCND1*‐mut introduced into HEK‐293 cells co‐transfected with miR‐338‐3p mimics or control using Lipofectamine 2000 reagent. Luciferase activities were normalized against Renilla luciferase.

### 2.9 Cell migration assay

Cell migration rate was measured by Transwell chamber assay (8 µm; Millipore, USA). In brief, FTC‐133 and B‐CPAP cells were digested and resuspended after transfection. A total of 2 × 10^5^ cells in about 250 μL serum‐free medium were added to the upper of transwell plates (Sigma‐Aldrich, St. Louis, MO). In addition, total volume of 500 μL medium was added into the lower chamber. Cultivated for 24 hours, cells on the upper chamber were washed by PBS, whereas migrated cells in the lower chamber were washed and fixed with formaldehyde for about 25 minutes. Optical microscope was applied for cell counting.

### 2.10 Flow cytometry analysis

Cell apoptosis status was determined following protocol of Annexin V‐FITC/PI‐cell apoptosis Detection Kit (Biolegend, San Diego, CA). After successively staining with both Annexin V‐FITC and protease inhibitors for half an hour, cells were incubated in a dark room for 25 minutes. Cell apoptotic status was analysed using MoFlow flow cytometer in whole experimental process.

### 2.11 RNA immunoprecipitation

RNA immunoprecipitation (RIP) assays were carried out using EZ‐Magna RIP RNA‐binding Protein Immunoprecipitation Kit (Millipore) following standard protocol. FTC‐133 cells were lysed using RIP lysis buffer, and then cell lysates were incubated with RIP buffer, including magnetic beads conjugated to human anti‐Ago2 antibody (Abcam), mouse IgG (Beyotime, China) served as control. Co‐precipitated RNAs were isolated using TRIzol reagent (TaKaRa, China). Ago‐IPs in this study was detected at least three times.

### 2.12 Tumour xenograft assay

All of the experimental protocols were permitted by Animal Care and Use Committee of China‐Japan Union Hospital of Jilin University and strictly followed the Guidelines for the Care and Use of Laboratory Animals by National Institute of Health. Twelve bagg albino, laboratory‐bred strain of the house mouse/c nude mice were provided by Charles River Laboratories (China). Cell suspension was injected subcutaneously into 8‐week‐old mice. Twelve mice were randomly separated into two groups. Tumour volumes were monitored weekly. Six weeks later, mice were killed using CO_2_ inhalation. Thereafter, tumour tissues were then collected and weighed. Tumour volume was measured every 7 days (tumour volume = 0.5 × length × width^2^).

### 2.13 Immunohistochemistry

Tissue sections were dried at 60°C for 1 hour and de‐waxed by automated dyeing machine and rehydrated. After washing with PBS, it was incubated with 3% hydrogen peroxide for about 5 minutes. Subsequently, the sections were immersed in 0.01 mol/L 3% citrate buffer and heated at 95°C in the microwave for quarter. After 30 minutes, non‐immune goat serum was added and sections were incubated overnight with Ki‐67 antibody from Abcam (Ab15580; Abcam) (1:300 v/v) at 4°C. After incubation with Ki‐67 antibody, sections should be washed in PBS and then incubated with HRP‐labelled goat anti‐rabbit IgG (Abcam) (1:1000 v/v) with sustaining for 30 minutes. Thereafter, section will be exposed to a freshly prepared diaminobenzidine for 4‐6 minutes and stained with hematoxylin for 15 seconds.

### 2.14 Statistical analysis

Data were presented as mean value ± standard deviation (Mean ± SD) in this study. GraphPad Prism 6.0 (La Jolla, CA) was recruited to conduct statistical analysis. Multiple comparisons were conducted by the one‐way ANOVA test. *P* value less than 0.05 was thought to be statistically significant.

## 3 RESULTS

### 3.1 The expression of both NR2F1‐AS1 and *CCND1* was higher in TC tissues than normal tissues based on microarray analysis

**Figure 1:**
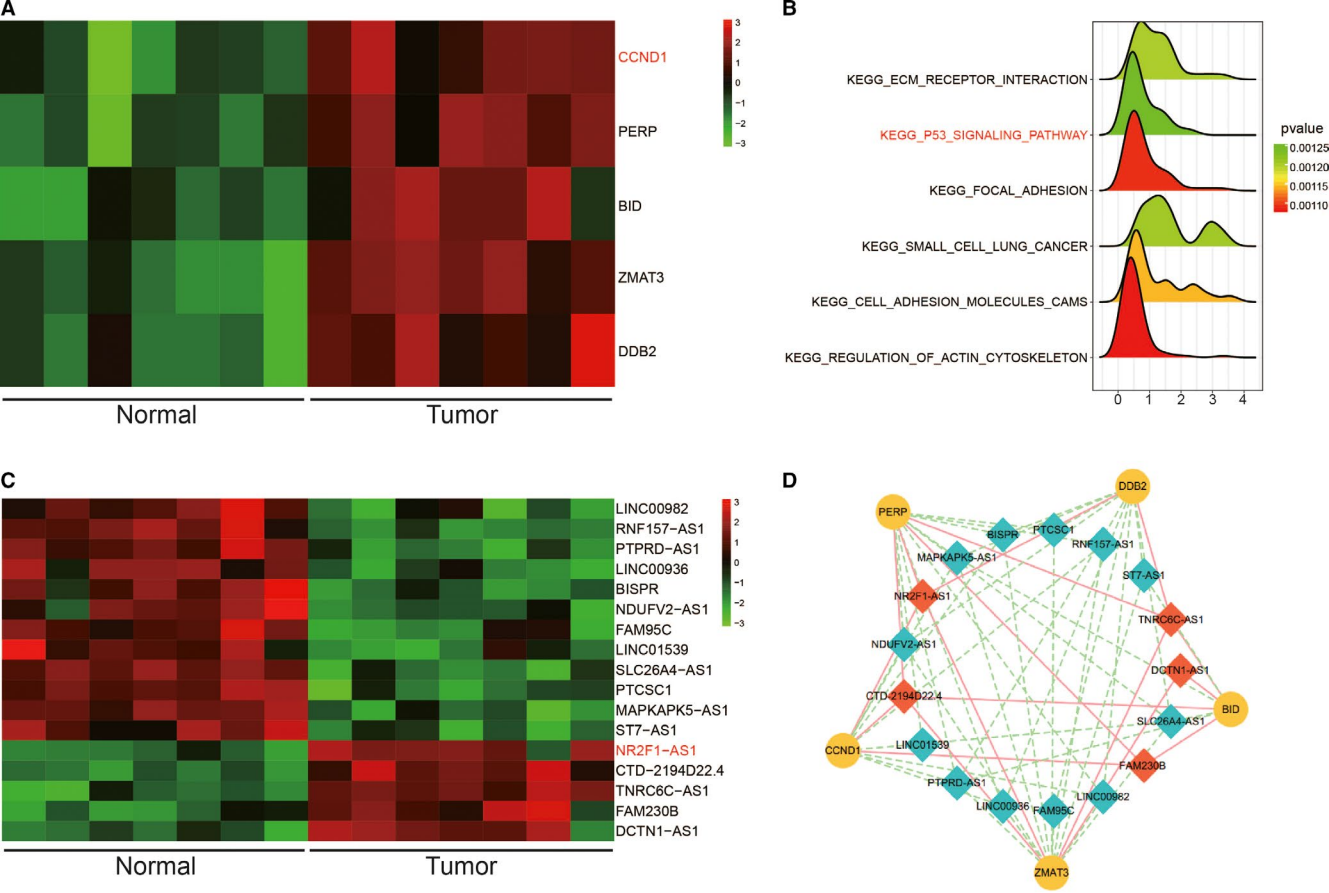
A, Visualization of top five up‐regulated mRNAs on p53 pathway in thyroid cancer (TC) tissues by heatmap; (B) kyoto encyclopedia of genes and genomes enrichment analysis revealed up‐regulated signalling pathways in TC tissues; (C) Visualization of top 17 differentially expressed lncRNA in TC tissues by heatmap; (D) Co‐expression network analysis of lncRNAs and mRNAs

It was reported that P53 signalling pathway played vital roles in tumour progression. Differentially expressed mRNAs in p53 pathway in TC tissues were selected out and visualized in Figure 1A. Among them, *CCND1* showed a robust increase in TC tissues by contrast with adjacent normal tissues, which indicated its potential oncogenic role in TC. P53 signalling pathway was greatly up‐regulated in TC as revealed in Figure 1B. Among differentially expressed lncRNAs, NR2F1‐AS1 was significantly were higher in TC tissues in Figure 1C. According to co‐expression network analysis, *CCND1* was predicted to be co‐expressed with NR2F1‐AS1 (Figure 1D). In short, the expression of both NR2F1‐AS1 and *CCND1* was higher in TC tissues than that in normal tissues based on microarray data.

### 3.2 Knockdown of NR2F‐AS1 suppressed cell proliferation and migration yet enhanced cell apoptosis in TC

**Figure 2:**
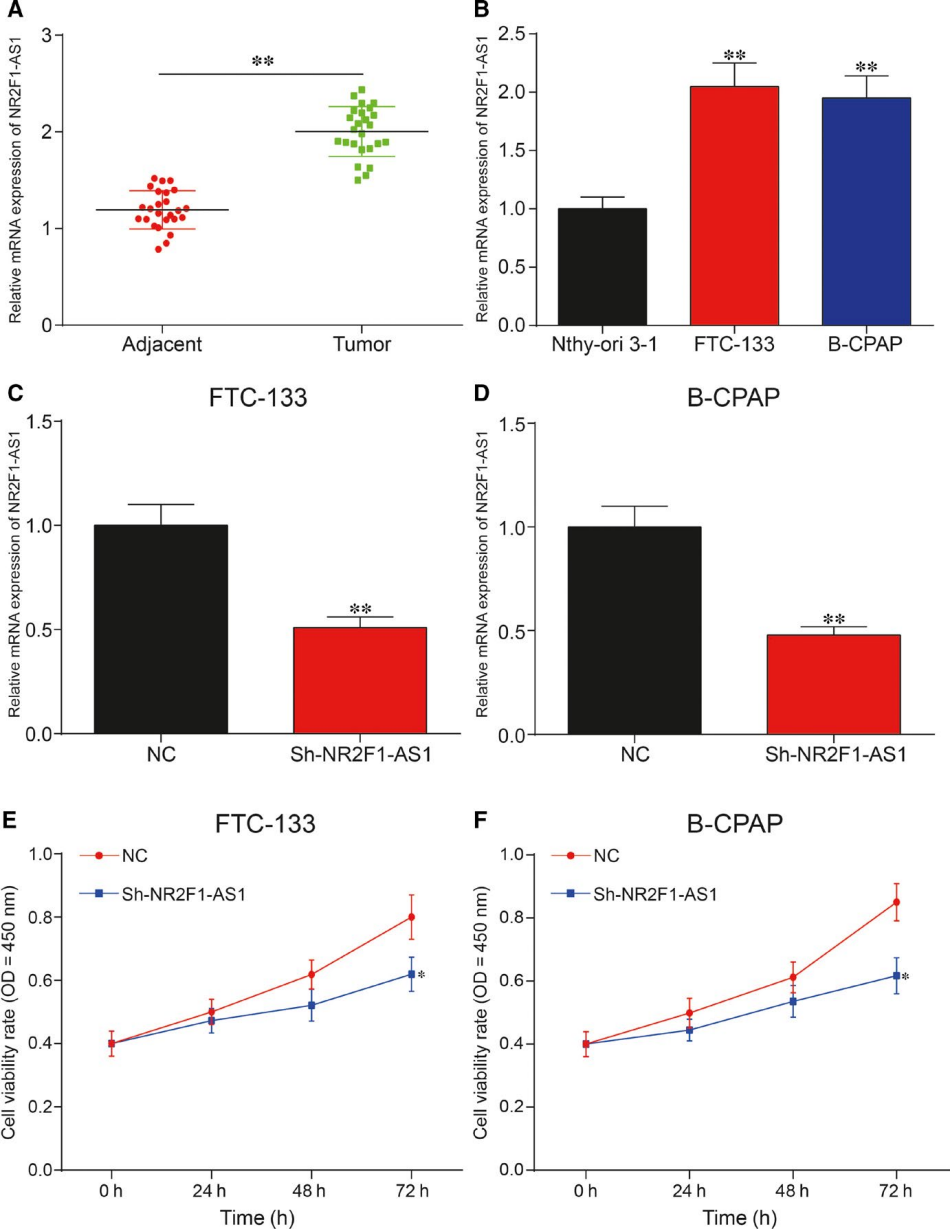
A, qRT‐PCR result showed that NR2F1‐AS1 was significantly increased in thyroid cancer (TC) tissues compared with adjacent normal tissues; (B) The expression of NR2F1‐AS1 was significantly up‐regulated in TC cell lines, FTC‐133 and B‐CPAP, compared with normal human thyroid cell line Nthy‐ori 3‐1; (C,D) Silence of NR2F1‐AS1 significantly down‐regulated the expression of NR2F1‐AS1 in FTC‐133 cells and B‐CPAP cells compared with negative control (NC) group, which indicated that the transfection in this study was successful; (E,F) Knockdown of NR2F1‐AS1 significantly suppressed cell viability rate in FTC‐133 cells and B‐CPAP cells compared with NC group. *P < 0.05, **P < 0.01

**Figure 3:**
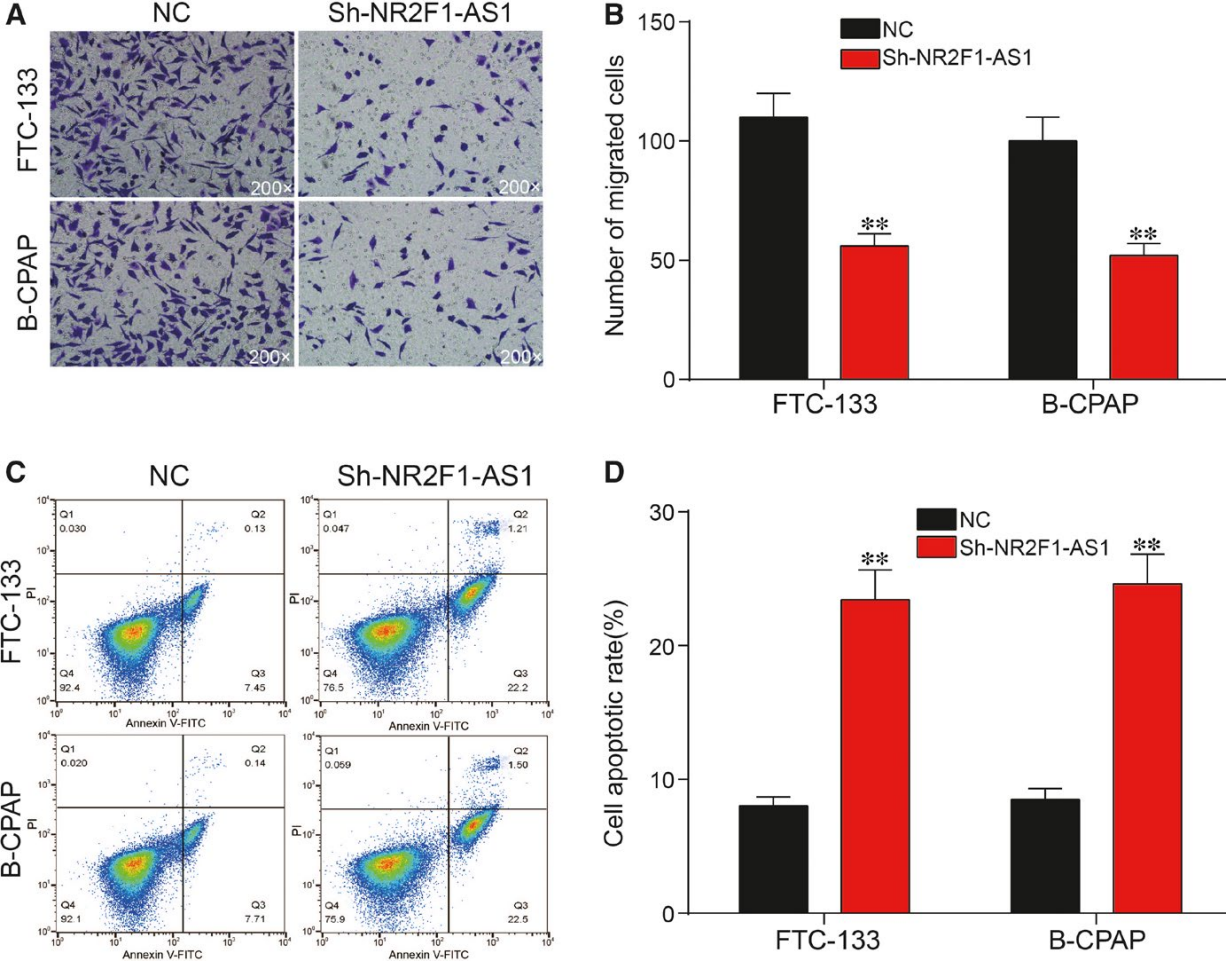
(A,B) Cell migration assay showed that silence of NR2F1‐AS1 could greatly inhibit cell migration in FTC‐133 cells and B‐CPAP cells compared with negative control (NC) group; (C,D) Flow cytometry assay showed that silence of NR2F1‐AS1 could greatly increase cell apoptosis in both FTC‐133 cells and B‐CPAP cells compared with NC group. **P < 0.01

After inspecting 25 TC tissues and paired adjacent normal tissues, NR2F‐AS1 was greatly increased in TC tissues (Figure 2A; *P* < 0.01). Besides, the result of qRT‐PCR showed that NR2F‐AS1 was up‐regulated in FTC‐133 cells and B‐CPAP cells compared with Nthy‐ori 3‐1 (Figure 2B; *P* < 0.01). qRT‐PCR was adopted to detect the expression of NR2F‐AS1 after transfection. As shown in Figure S1, plasmid construction was successful. The results revealed that NR2F‐AS1 was decreased obviously after transfection (Figure 2C,D; *P* < 0.01), indicating successful transfection. The results of CCK‐8 assay showed that cell viability rate was tremendously lower in TC cells with knockdown of NR2F‐AS1 than in NC group (Figure 2E,F; *P* < 0.05). Cell migration assay results showed that the migration ability of cells in sh‐NR2F‐AS1 group was significantly suppressed in TC cells (Figure 3A,B; *P* < 0.01). In brief, silence of NR2F‐AS1 greatly decreased cell viability rate and cell migration rate in TC cells. In addition, flow cytometry results indicated that suppression of NR2F‐AS1 could largely increase cell apoptotic rate in both FTC‐133 cells and B‐CPAP cells compared with NC group (Figure 3C,D; *P* < 0.01). Taken together, down‐regulation of NR2F‐AS1 could inhibit cell viability and migration, whereas enhance cell apoptosis in TC.

### 3.3 miR‐338‐3p shared a targeted relationship with NR2F1‐AS1 and was down‐regulated in TC

**Figure 4:**
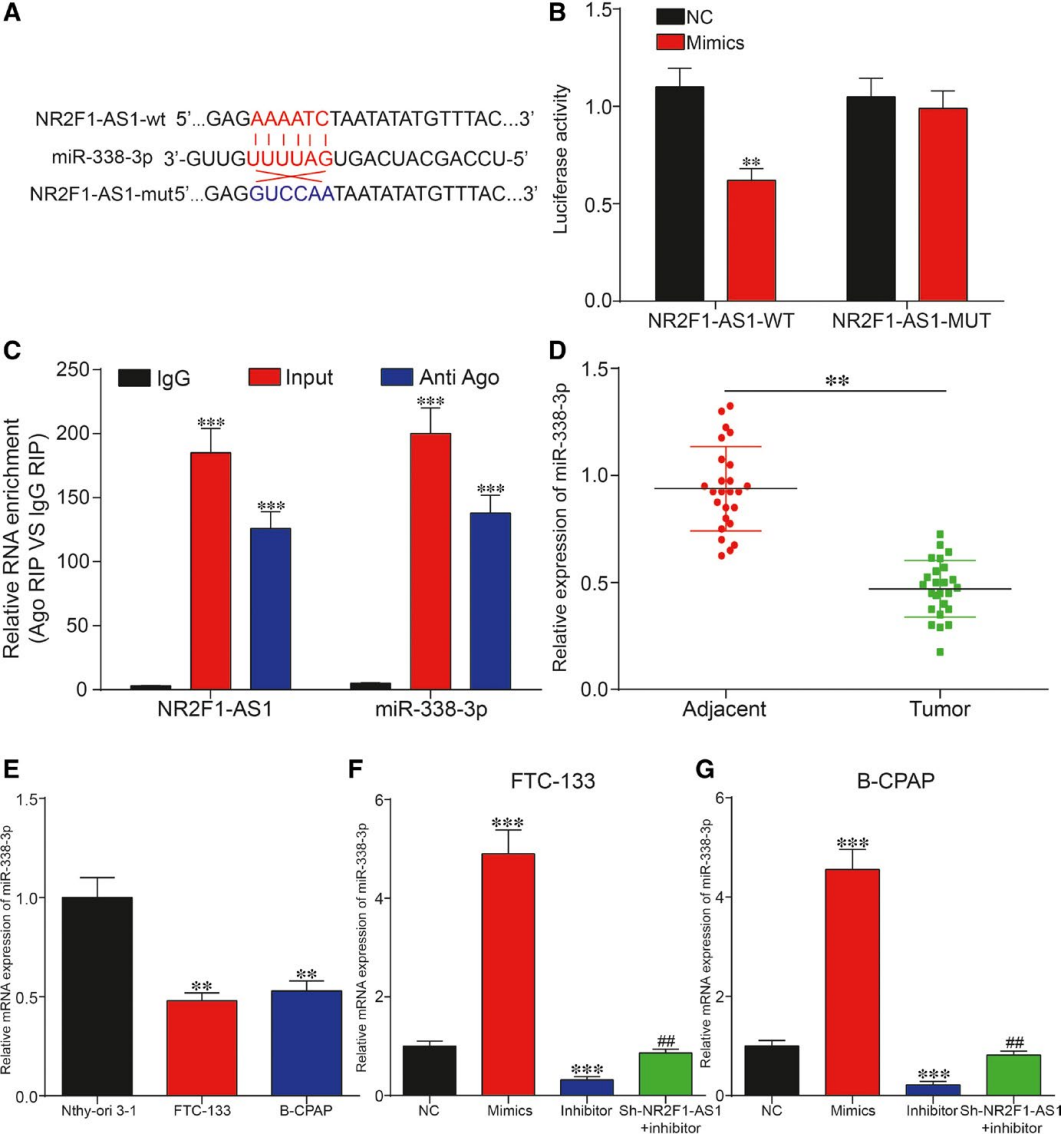
A, Predicted binding site between NR2F1‐AS1 and miRNA‐338‐3p by miRcode; (B,C) Dual‐luciferase reporter gene assay and RNA immunoprecipitation assay confirmed the targeted relationship between NR2F1‐AS1 and miR‐338‐3p; (D) miR‐338‐3p was down‐regulated in thyroid cancer (TC) tissues compared with adjacent normal tissues; (E) miR‐338‐3p was significantly decreased in TC cell lines compared with Nthy‐ori 3‐1; (F,G) miR‐338‐3p mimics increased the expression of miR‐338‐3p, which was otherwise decreased by miR‐338‐3p inhibitor compared with negative control (NC) group. **P < 0.01, compared with NC group; ***P < 0.001, compared with lgG group; ^##^ *P* < 0.01, compared with inhibitor group

According to miRcode results, miR‐338‐3p was predicted to bind to the 3′‐UTR of NR2F1‐AS1‐wt as shown in Figure 4A. Hence, dual‐luciferase reporter assay was employed for further verification of the targeted relationship between miR‐338‐3p and NR2F1‐AS1. It was demonstrated that relative luciferase activity in group co‐transfected with miR‐338‐3p mimics and NR2F1‐AS1‐wt showed lower level than that of group with co‐transfection of both NR2F1‐AS1‐wt and NC in Figure 4B, whereas there was no significant change in NR2F1‐AS1‐mut group, revealing that there was the direct binding between miR‐338‐3p and NR2F1‐AS1. As shown in Figure 4C, RNA was significantly more enriched in the Ago‐IP fractions of FTC‐133 cells compared with IgG‐IP group (*P* < 0.001), indicating the direct targeted relationship between NR2F1‐AS1 and miR‐338‐3p. Thereafter, miR‐338‐3p expression level was determined via qRT‐PCR in the 25 paired tissues. qRT‐PCR results showed that miR‐338‐3p was greatly down‐regulated in TC tissues (Figure 4D; *P* < 0.01). Similarly, the result of qRT‐PCR showed that miR‐338‐3p expression was markedly lower in FTC‐133 cells and B‐CPAP cells than in Nthy‐ori 3‐1 cells (Figure 4E; *P* < 0.01). In addition, qRT‐PCR was employed to verify the relative expression of miR‐338‐3p after transfection. The results revealed that miR‐338‐3p inhibitor greatly down‐regulated the expression of miR‐338‐3p, which was instead up‐regulated by miR‐338‐3p mimics (Figure 4F,G; *P* < 0.001). Besides, miR‐338‐3p inhibitor decreased the expression of miR‐338‐3p in FTC‐133 cells and B‐CPAP cells, which could be retrieved to normal expression level with down‐regulation of NR2F1‐AS1 (*P* < 0.01). In short, miR‐338‐3p was validated to directly target NR2F1‐AS1 and down‐regulated in TC cells.

### 3.4 NR2F1‐AS1 promoted cell proliferation and cell migration yet suppressed cell apoptosis by down‐regulating miR‐338‐3p in TC

**Figure 5:**
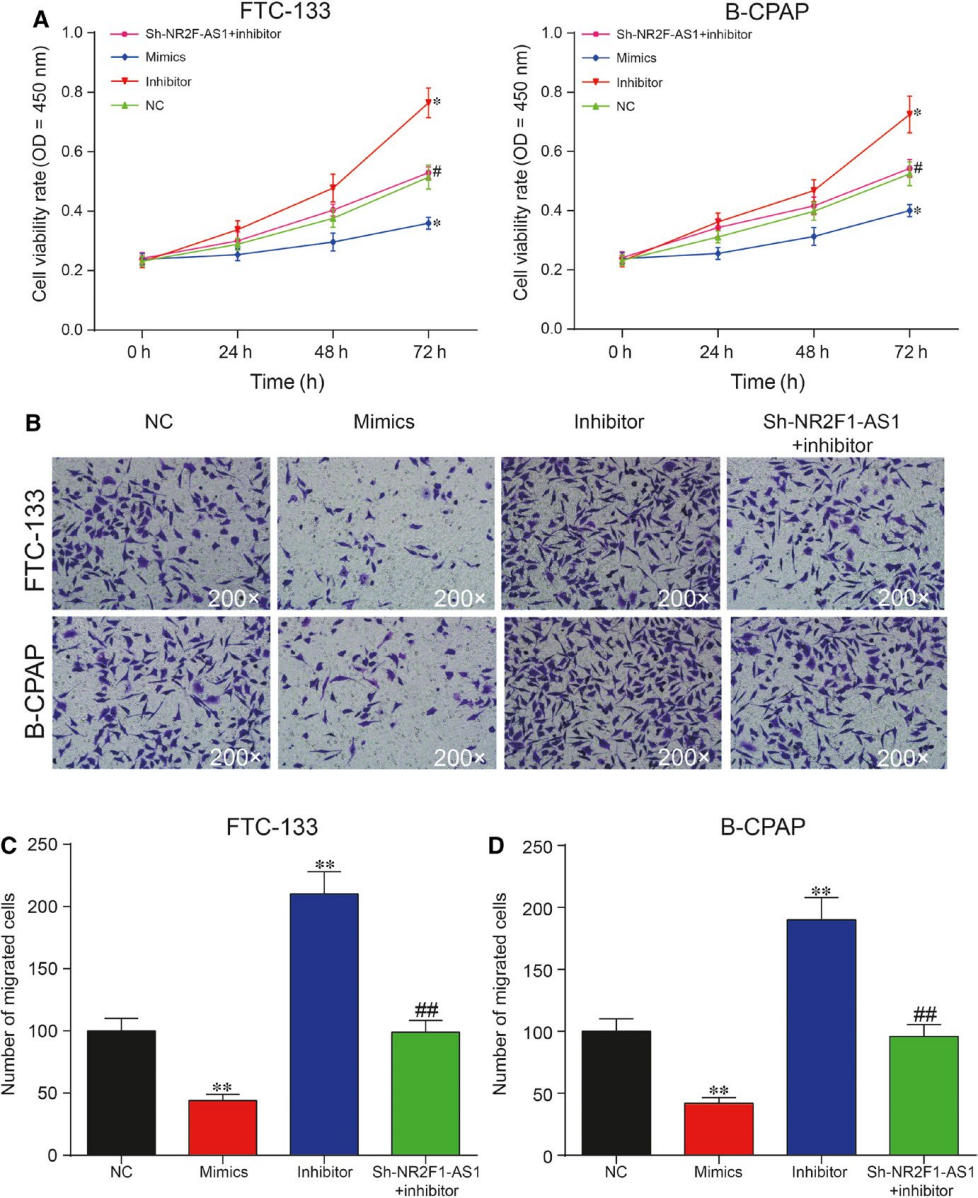
A, Overexpression of miR‐338‐3p greatly suppressed proliferation, which was otherwise promoted by inhibition of miR‐338‐3p compared with negative control (NC) group; (B,C) Cell migration assay showed that overexpression of miR‐338‐3p greatly suppress migration, which was otherwise promoted by suppression of miR‐338‐3p compared with NC group. *P < 0.05, **P < 0.01, compared with NC group; ^#^ *P* < 0.05, ^##^ *P* < 0.01, compared with inhibitor group

**Figure 6:**
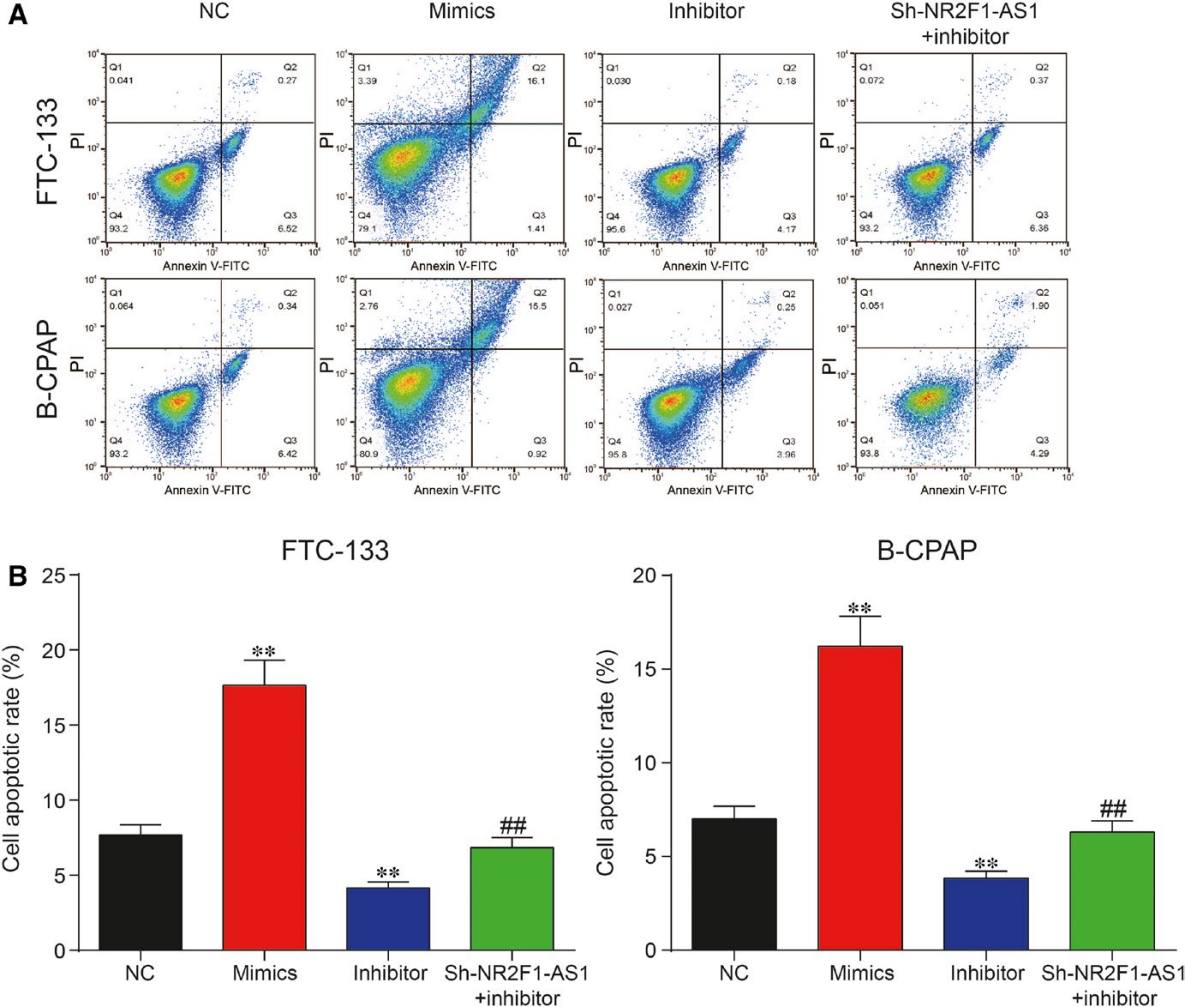
(A,B) Cell Counting Kit‐8 assay showed that overexpression of miR‐338‐3p greatly increased cell apoptosis rate in FTC‐133 cells and B‐CPAP cells, which could be otherwise reversed with inhibition of miR‐338‐3p. **P < 0.01, compared with negative control (NC) group; ^##^ *P* < 0.01, compared with inhibitor group

To unveil the roles of miR‐338‐3p/NR2F1‐AS1 axis in TC, both CCK‐8 assay and cell migration assay were adopted to assess cell proliferation rate and cell migration rate, respectively. It was unveiled by CCK‐8 assay that increase in miR‐338‐3p could suppress cell proliferation, whereas inhibition of miR‐338‐3p could otherwise enhance cell proliferation in FTC‐133 cells and B‐CPAP cells (Figure 5A; *P* < 0.05). The result of cell migration assay indicated that overexpression of miR‐338‐3p could greatly suppress cell migration, which was instead enhanced with the inhibition of miR‐338‐3p in both FTC‐133 cells and B‐CPAP cells (Figure 5B,C; *P* < 0.01). Furthermore, flow cytometry assay showed that overexpression of miR‐338‐3p led to an increase in cell apoptotic rate, whereas down‐regulation of miR‐338‐3p reduced cell apoptotic rate in both FTC‐133 cells and B‐CPAP cells. In addition, miR‐338‐3p inhibitor decreased cell apoptotic rate in both cell lines, which could be recovered to normal level with co‐transfection with sh‐NR2F1‐AS1 (Figure 6A,B; *P* < 0.01). Taken together, NR2F1‐AS1 promoted proliferation and migration yet suppressed apoptosis of TC cells by down‐regulation of miR‐338‐3p.

### 3.5 *CCND1* was directly targeted by miR‐338‐3p and could be regulated by NR2F1‐AS1/miR‐338‐3p in TC

**Figure 7:**
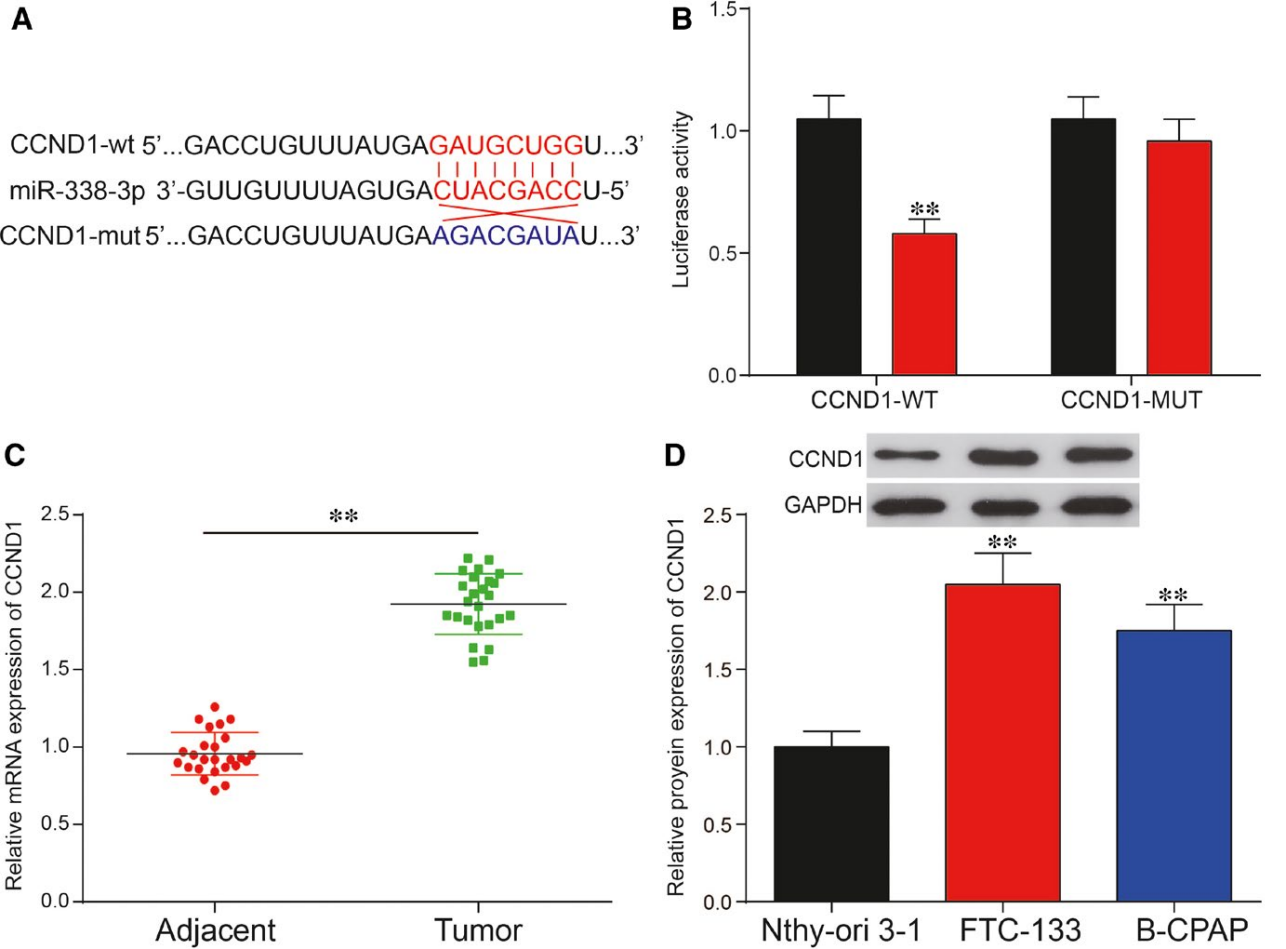
A, Predicted binding site between Cyclin D1 (*CCND1*) and miRNA‐338‐3p by TargetScanHuman; (B) Dual‐luciferase reporter assay confirmed the targeted relationship between *CCND1* and miR‐338‐3p; (C) *CCND1* was significantly up‐regulated in thyroid cancer (TC) tissues compared with adjacent normal tissues. D, *CCND1* was significantly increased in TC cell lines, FTC‐133 and B‐CPAP compared with normal human thyroid cell line Nthy‐ori 3‐1. **P < 0.01

**Figure 8:**
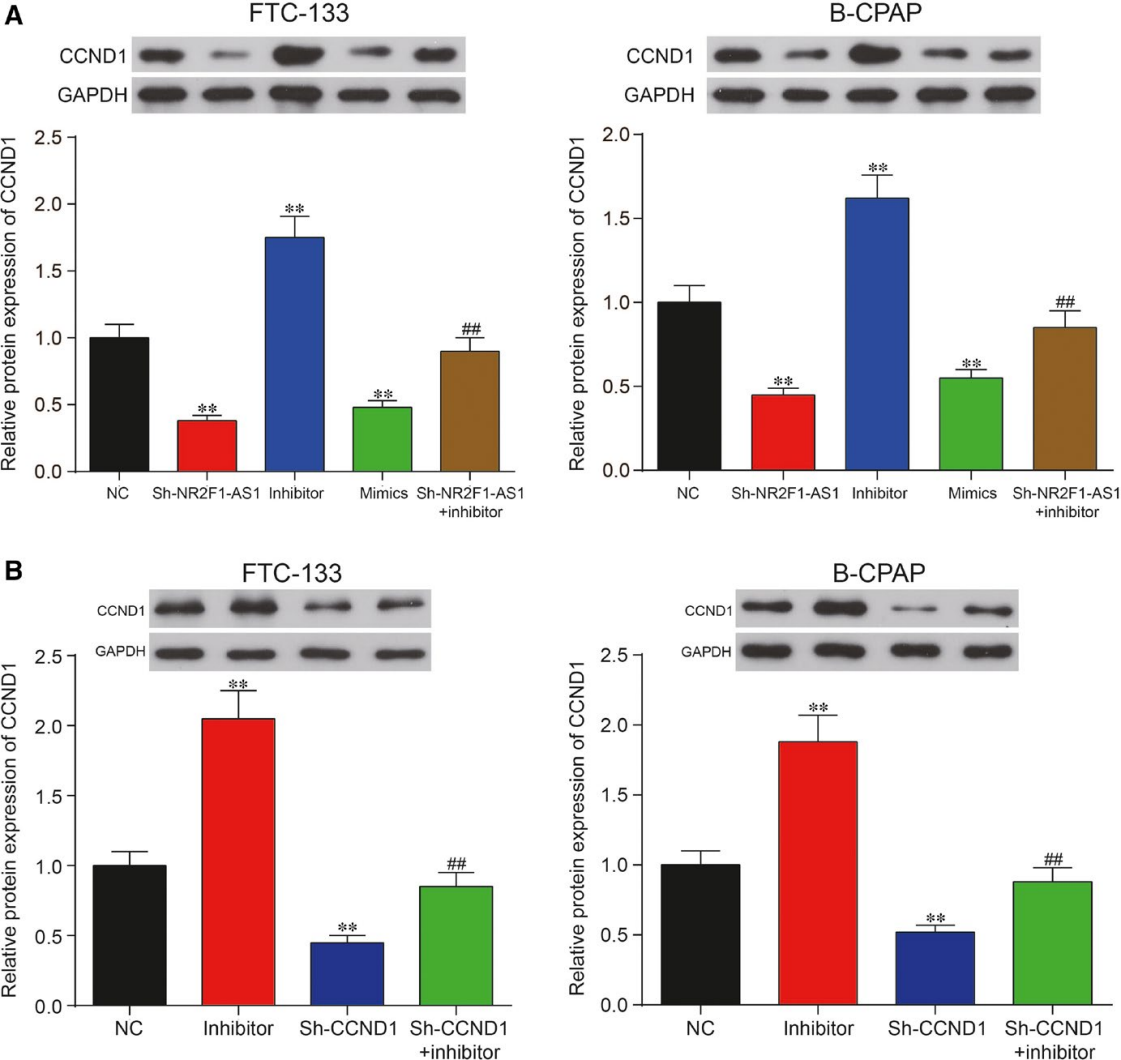
A, miR‐338‐3p mimics or silence of NR2F1‐AS1 decreased the protein expression of cyclin D1 (*CCND1*), which was instead increased by miR‐338‐3p inhibitor compared with negative control (NC) group in FTC‐133 cells and in B‐CPAP cells. B, Sh‐*CCND1* significantly inhibited *CCND1* protein expression, which was recovered to the normal level by co‐transfection with miR‐338‐3p inhibitor in FTC‐133 cells and B‐CPAP cells. **P < 0.01, compared with NC group; ^##^ *P* < 0.01, compared with inhibitor group

The results of bioinformatic analysis based on TargetScan revealed that *CCND1* might be the target of miR‐338‐3p (Figure 7A). To validate the targeted relationship between miR‐338‐3p and *CCND1*, dual‐luciferase reporter gene assay was utilized. Relative luciferase activity in that group co‐transfected with miR‐338‐3p mimics and *CCND1*‐wt revealed lower level than that of group with co‐transfection of both miR‐338‐3p NC and *CCND1*‐wt, suggesting the direct binding between miR‐338‐3p and *CCND1* in TC (Figure 7B). Afterwards, the relative mRNA expression level of *CCND1* was measured by qRT‐PCR in the 25 paired tissues. The result of qRT‐PCR revealed significant up‐regulation of *CCND1* in the TC tissues (Figure 7C; *P* < 0.01). Similarly, up‐regulation of C*CND1* was also observed in TC cell lines in contrast with normal thyroid cell line. The results of Western blot analysis showed that *CCND1* protein expression was overexpressed in FTC‐133 cells and B‐CPAP cells compared with Nthy‐ori 3‐1 cell (Figure 7D; *P* < 0.01). Subsequently, Western blot analysis was then employed to further uncover the regulation of miR‐338‐3p/NR2F1‐AS1 on CCND1 protein expression in TC. Overexpression of miR‐338‐3p or silence of NR2F1‐AS1 significantly suppressed CCND1 protein expression, whereas inhibition of miR‐338‐3p otherwise up‐regulated CCND1 protein expression compared with the NC group in FTC‐133 cells and B‐CPAP cells (Figure 8A; *P* < 0.01). Besides, knockdown of NR2F1‐AS1 greatly decreased *CCND1* protein expression, which could be retrieved to normal levels co‐transfected with miR‐338‐3p inhibitor. In addition, Figure 8B revealed that sh‐*CCND1* significantly down‐regulated *CCND1* protein expression, which was recovered to the normal expression level by co‐transfection with miR‐338‐3p inhibitor in both cell lines. In short, *CCND1* was targeted by miR‐338‐3p and could be regulated by NR2F1‐AS1/miR‐338‐3p in TC.

### 3.6 miR‐338‐3p suppressed proliferation and migration, whereas enhanced apoptosis of TC cells via down‐regulation of ***CCND1***

**Figure 9:**
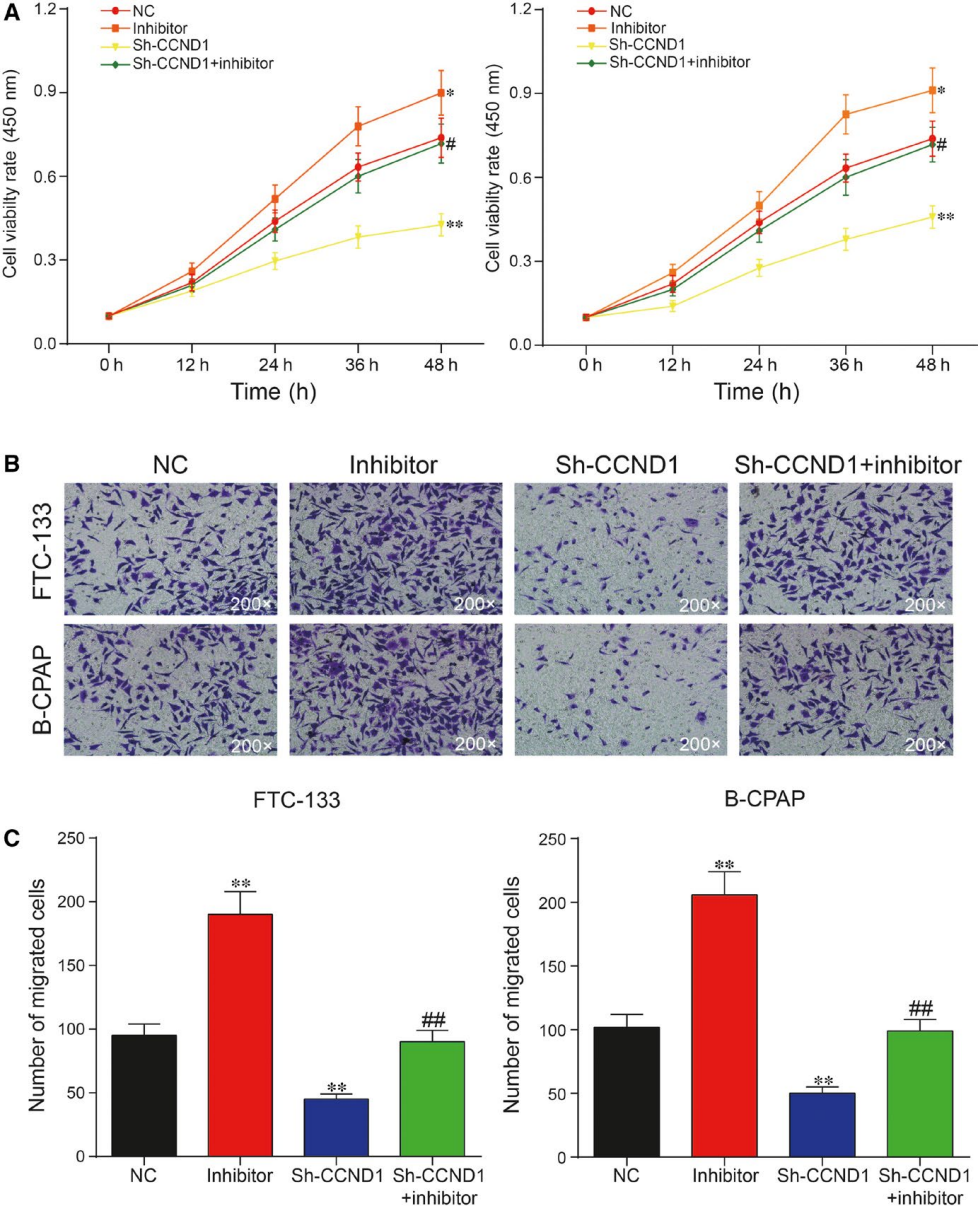
A, Down‐regulation of cyclin D1 (*CCND1*) greatly suppressed cell proliferation, which was otherwise up‐regulated by miR‐338‐3p inhibitor in both FTC‐133 cells and B‐CPAP cells compared with negative control (NC) group; (B,C) Cell migration assay showed that knock‐down of *CCND1* greatly inhibited cell migration, which was instead promoted with miR‐338‐3p inhibitor in both FTC‐133 cells and B‐CPAP cells compared with NC group. **P < 0.01, compared with NC group; ^##^ *P* < 0.01, compared with inhibitor group

**Figure 10:**
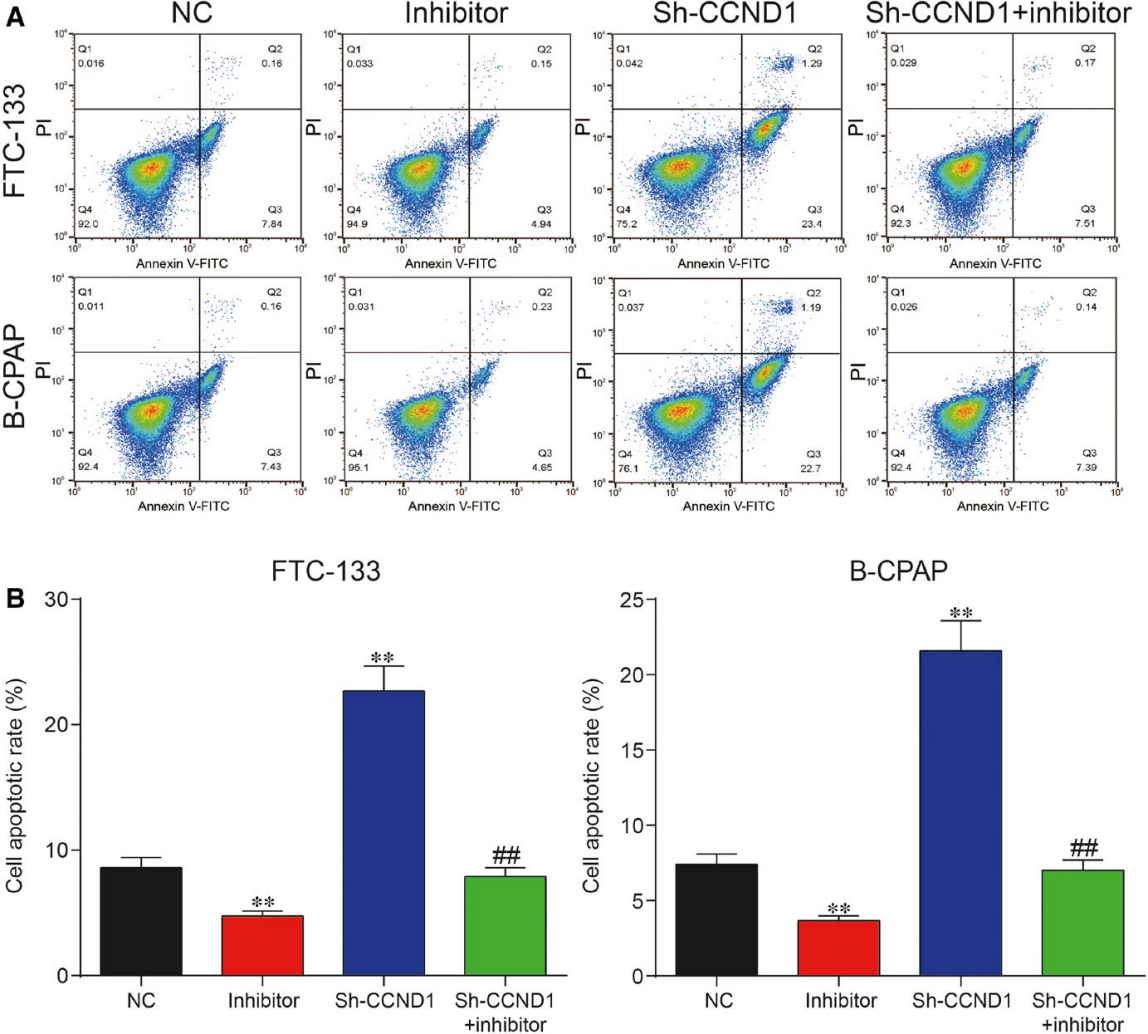
(A,B) Flow cytometry assay showed that down‐regulation of cyclin D1 (*CCND1*) significantly increased apoptotic rate, which could be otherwise decreased with miR‐338‐3p inhibitor in both FTC‐133 cells and B‐CPAP cells in comparison with the negative control (NC) group. **P < 0.01, compared with NC group; ^##^ *P* < 0.01, compared with inhibitor group

The results of CCK‐8 assay revealed that inhibition of *CCND1* greatly suppressed cell proliferation, whereas suppression of miR‐338‐3p largely enhanced cell proliferation in both FTC‐133 cells and B‐CPAP cells in Figure 9A. In addition, cell migration assay showed that suppression of *CCND1* inhibited cell migration, yet inhibition of miR‐338‐3p promoted cell migration in both FTC‐133 cells and B‐CPAP cells (Figure 9B,C; *P* < 0.01). In addition, flow cytometry assay showed that inhibition of *CCND1* significantly increased apoptotic rate (Figure 10A,B; *P* < 0.01), which was instead reversed by down‐regulation of miR‐338‐3p in both FTC‐133 cells and B‐CPAP cells. To summarize, miR‐338‐3p suppressed proliferation and migration, whereas promoted apoptosis of TC cells by down‐regulation of *CCND1*.

### 3.7 Silence of NR2F1‐AS1 suppressed TC tumorigenesis in vivo

**Figure 11:**
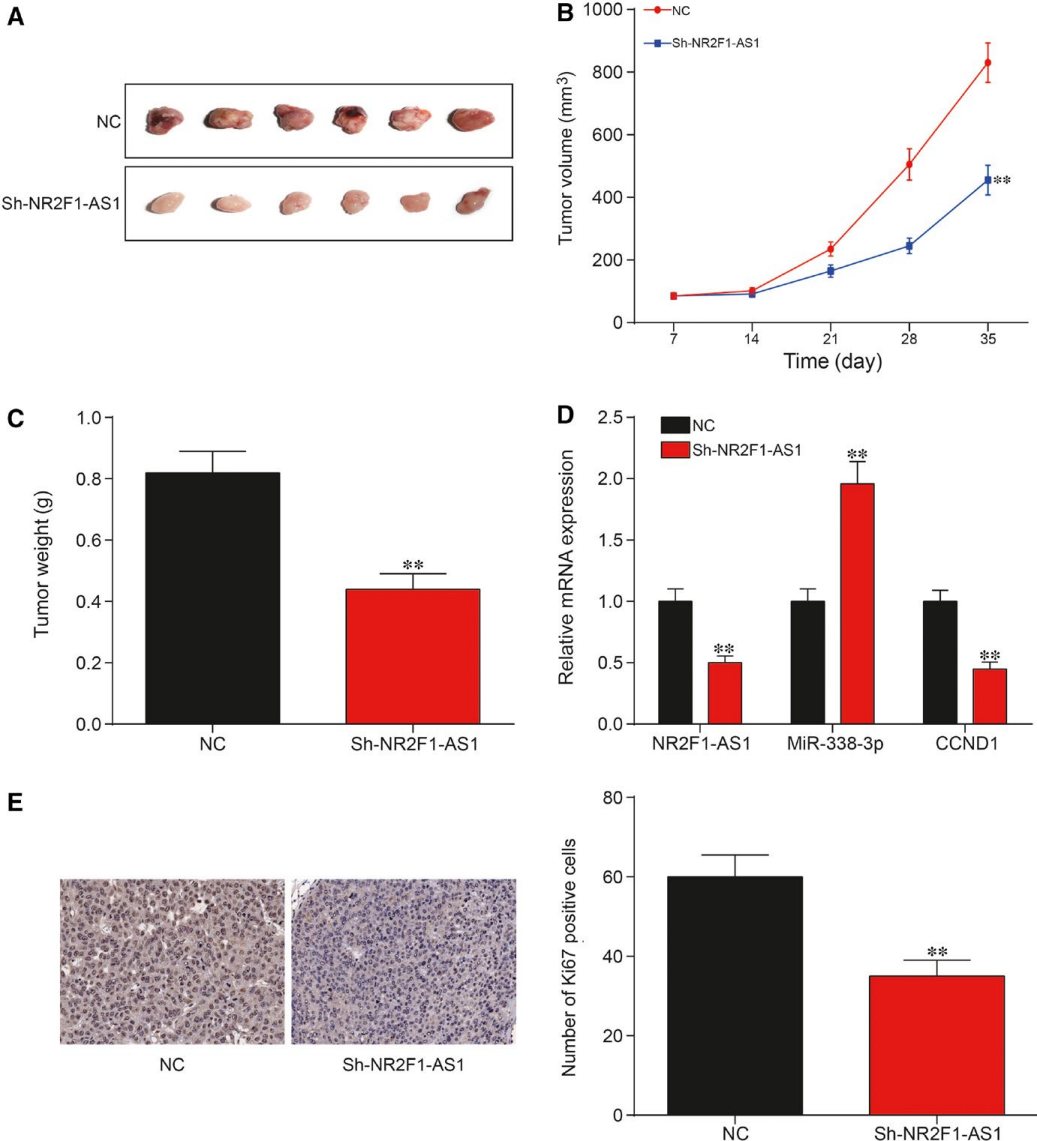
(A‐C) As evidenced by decrease in tumour volume and weight, suppression of NR2F1‐AS1 could significantly suppress thyroid cancer (TC) tumorigenesis in vivo; (D) The detection of expression levels of NR2F1‐AS1, miR‐338‐3p and cyclin D1 (*CCND1*) with silence of NR2F1‐AS1; (E) Immunohistochemistry result showed that down‐regulation of NR2F1‐AS1 suppressed TC tumorigenesis in vivo as revealed by change of positive cells, **P < 0.01

To further explore the role of NR2F1‐AS1 in TC progression in vivo, xenograft nude mouse model was recruited to evaluate its function and FTC‐133 cell line was adopted in in vivo study. Xenograft mice with the knockdown of NR2F1‐AS1 displayed a significant decrease in tumour weight and tumour volume compared with NC groups 5 weeks after injection (Figure 11A‐C; *P* < 0.01). qRT‐PCR result revealed that the expression levels of both NR2F1‐AS1 and *CCND1* were decreased whereas miR‐338‐3p was up‐regulated after down‐regulation of NR2F1‐AS1, which was in line with result from in vitro experiments (Figure 11D; *P* < 0.01). In addition, immunohistochemistry (IHC) result showed that down‐regulation of NR2F1‐AS1 suppressed TC tumorigenesis in vivo as revealed by decrease in number of positive cells as shown in Figure 11E. In short, knockdown of NR2F1‐AS1 inhibited TC tumorigenesis in vivo.

## 4 DISCUSSION

In our study, NR2F1‐AS1 and *CCND1* was greatly up‐regulated, whereas miRNA‐338‐3p was otherwise suppressed in TC tissues and cell lines compared with adjacent normal tissues and cell lines. Down‐regulation of NR2F1‐AS1 inhibited proliferation and migration, whereas enhanced apoptosis of TC cells by decreasing the expression of miRNA‐338‐3p yet increasing the expression of *CCND1*. In brief, these findings will provide us with more significant contributions to further treatment of TC.

Long non‐coding RNAs, as well as lncRNA/miRNA axis always played pivotal roles in the initiation and progression of a set of cancers. Previous report showed that lncRNA NEAT1 regulated TC progression by modulating miR‐129‐5p expression.^27^ Besides, lncRNA CCAT1 promoted cell growth and cell migration by down‐regulation of miR‐143 in TC cell line FTC‐133.^28^ Furthermore, lncRNA TUG1 influenced papillary thyroid cancer proliferation, migration and EMT formation through targeting miR‐145.^29^ In addition, NR2F1‐AS1 regulated hepatocellular carcinoma resistance by regulating the expression of miR‐363.^8^ Our result revealed that the expression of NR2F1‐AS1 was higher in TC tissues and cell lines than adjacent normal tissues and cell lines. In addition, NR2F1‐AS1 promoted TC cell proliferation and migration yet suppressed cell apoptosis by down‐regulating miR‐338‐3p expression, which was in line with suppressor role of miR‐338‐3p in TC.^30^

miRNAs always governed biological processes by repressing translation progress or triggering the degradation of its target mRNAs.^31^ Furthermore, previous studies showed that miRNAs played pivotal roles in the treatment of tumours.^32^ Our results showed that miR‐338‐3p was down‐regulated in TC tissues and cell lines in comparison with adjacent normal tissues and cell lines, indicating that miR‐338‐3p might well function as tumour suppressor to affect TC progression. Several studies revealed the significance of miRNAs in TC. miR‐214 regulated papillary thyroid carcinoma (PTC) cell metastasis and growth by regulating *PSMD10*.^33^ In addition, miRNA‐361‐5p inhibited PTC progression by targeting *ROCK1*. However, the roles of miR‐338‐3p in TC progression, especially the relationship with mRNA were still poorly understood. miR‐338‐3p was reported to function as a tumour suppressor to inhibit renal cell carcinoma progression.^34^ miR‐338‐3p suppressed growth and invasion of non‐small cell lung cancer cells by targeting *IRSA2*.^35^ Besides, miR‐338‐3p inhibited TC progression through targeting *AKT3*.^30^ Our findings revealed that miR‐338‐3p suppressed cell proliferation and migration, whereas enhanced cell apoptosis in TC by targeting *CCND1*, which is similar with the roles of miR‐338‐3p/*CCND1* axis in HCC.^36^

Cyclin D1, a member of the family of cyclins, can control the passage of proliferating cells through cell cycle.^37^ Up‐regulation of *CCND1* was observed in both benign and malignant thyroid tumours.^37^ Dys‐regulation of cyclin is closely related with oncogenesis and *CCND1* functions as an oncogene in tumour progression. It was reported that *CCND1* could be directly targeted by miR‐186 to affect lung adenocarcinoma progression.^25^ In addition, previous report showed that *CCND1* could be directly targeted by miR‐195 to affect tumour growth and metastasis in PTC Cell Lines.^24^ Our results revealed that *CCND1* was greatly up‐regulated in TC tissues and cell lines. Besides, knock‐down of *CCND1* greatly suppressed cell proliferation and migration yet promoted cell apoptosis in TC, which was significant for the validation of the oncogenic roles of *CCND1* in TC. Besides, *CCND1* may well be selected as a potential therapeutic target for the TC.

However, the limitations of this study should not be overlooked. To begin with, the study population was not big enough for further validation of the great significance of NR2F1‐AS1/miRNA‐338‐3p*/CCND1* axis in the progression of TC. Besides, *CCND1* may be down‐stream of other miRNAs apart from miR‐338‐3p in TC. Similarly, miR‐338‐3p might well target other genes besides *CCND1* in TC. Furthermore, diagnosis and treatment of TC is far more sophisticated than our expectations. Therefore, it really does matter to identify new lncRNAs, miRNAs and mRNAs, as well as interpret the mechanisms to facilitate therapeutic development against TC.

Above all, NR2F1‐AS1 acted as a ceRNA of miR‐338‐3p and releasing *CCND1* to promote the development of TC, which might well aid intervention strategies of TC in the future.

## ETHICS APPROVAL AND CONSENT TO PARTICIPATE

The procedures in the study were scrutinized and approved by Medical Ethics Committee of China‐Japan Union Hospital of Jilin University and TC donors involved in the study signed their informed consent allowing the use of their tissue samples for scientific research. All of the experimental protocols were permitted by Animal Care and Use Committee of China‐Japan Union Hospital of Jilin University and strictly followed the Guidelines for the Care and Use of Laboratory Animals by National Institute of Health.

## CONFLICT OF INTERESTS

The authors declare that they have no conflicts of interest with the contents of this article.

## Data Availability

The data that support the findings of this study are available from the corresponding author upon reasonable request.
