# Supplementary material for: Long non‐coding RNA NR2F1‐AS1 promoted proliferation and migration yet suppressed apoptosis of thyroid cancer cells through regulating miRNA‐338‐3p/CCND1 axis
Source: J Cell Mol Med. 2019 Jul 14;23(9):5907–19. doi: 10.1111/jcmm.14386 (PMC6714216; doi:10.1111/jcmm.14386)
Supplement: Supplementary file 1 [file JCMM-23-5907-s001.docx]

**Figure S1** Figures of gel electrophoresis showed that plasmid construction was successful. 1. pENTR/U6 vector; 2. Linearized pENTR/U6 vector; 3.sh-NR2F1-AS1; 4. pENTR/U6-sh-NR2F1-AS1
