# Supplementary figures and images for: Long non‐coding RNA NR2F1‐AS1 promoted proliferation and migration yet suppressed apoptosis of thyroid cancer cells through regulating miRNA‐338‐3p/CCND1 axis
Source: J Cell Mol Med. 2019 Jul 14;23(9):5907–19. doi: 10.1111/jcmm.14386 (PMC6714216; doi:10.1111/jcmm.14386)

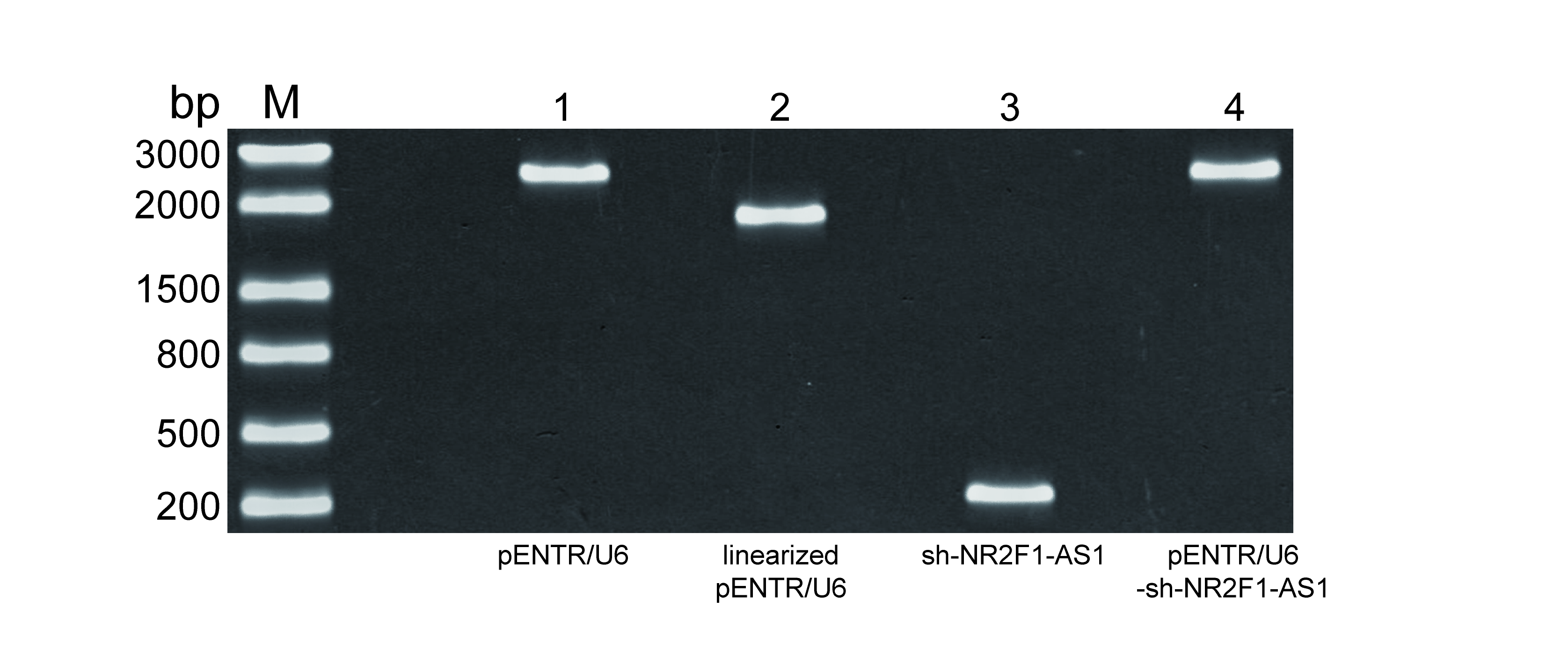

Supplement: Supplementary file 2 [file JCMM-23-5907-s002.tif]
